# Influence of Uncertainties in Optode Positions on Self-Calibrating or Dual-Slope Diffuse Optical Measurements

**DOI:** 10.3390/photonics12070697

**Published:** 2025-07-10

**Authors:** Giles Blaney, Angelo Sassaroli, Tapan Das, Sergio Fantini

**Affiliations:** 1 Department of Biomedical Engineering, Tufts University, 4 Colby Street, Medford MA 02155, USA; 2 Jorhat Institute of Science and Technology, Chenijaan, Jorhat 785010, Assam, India

**Keywords:** self-calibrating, dual-slope, optode geometry, tissue spectroscopy, absorption coefficient, reduced scattering coefficient

## Abstract

Self-calibrating and dual-slope measurements have been used in the field of diffuse optics for robust assessment of absolute values or temporal changes in the optical properties of highly scattering media and biological tissue. These measurements employ optical probes with a minimum of two source positions and a minimum of two detector positions. This work focuses on a quantitative analysis of the impact of errors in these source and detector positions on the assessment of optical properties. We considered linear, trapezoidal, and rectangular optode arrangements and theoretical computations based on diffusion theory for semi-infinite homogeneous media. We found that uncertainties in optodes’ positions may have a greater impact on measurements of absolute scattering versus absorption coefficients. For example, a 4.1% and 19% average error in absolute absorption and scattering, respectively, can be expected by displacing every optode in a linear arrangement by 1 mm in any direction. The impact of optode position errors is typically smaller for measurements of absorption changes. In each geometrical arrangement (linear, trapezoid, rectangular), we identify the direction of the position uncertainty for each optode that has minimal impact on the optical measurements. These results can guide the optimal design of optical probes for self-calibrating and dual-slope measurements.

## 1. Introduction

In Frequency-Domain (FD) Near-InfraRed Spectroscopy (NIRS) strategies for the retrieval of the bulk absolute optical properties of optically turbid media are based on multi-frequency [1] or multi-distance [2] measurements. In both cases a calibration of the experimental apparatus on a phantom of known optical properties is required. This is not a trivial step, and the success of the calibration procedure for the quantification of the absolute optical properties of a target medium is based on several assumptions: (1) that a calibration phantom of known (i.e., high accuracy) optical properties and robust procedures for its quantification exists [3]; (2) that the phantom is optically homogeneous; (3) that one is able to reproduce the optical coupling between the probe and the calibration phantom also during the experiment on a medium of unknown optical properties; (4) that the laser power and the detector gain are stable. These are not conditions that are always easy to satisfy. For example, experiments that last several hours might need recalibration of the system due to laser power and detector gain fluctuations. Ideally, pressure sensors to control the optical coupling might be needed. During in vivo experiments on human subjects, the requirement of stable coupling might be particularly difficult to achieve, due to uncontrolled subject motion. Therefore, methods that bypass the limiting factors of calibration and that provide accurate estimates of the optical properties are highly welcome.

Hueber et al. [4] first proposed the Self-Calibrating (SC) method for the retrieval of the absolute optical properties of tissues in FD NIRS (namely the absorption coefficient ($\mu_{a}$) and the reduced scattering coefficient ($\mu_{s}^{\prime }$). The proposed method provided a robust way to retrieve the optical properties with the advantage of being highly insensitive to the limiting factors intrinsic in a calibration procedure. Since then, the SC method has found a number of applications for the estimation of tissue oxygen Saturation (StO_2_) [5-7], for the measurement of absorption coefficient change ($\Delta \mu_{a}$) of target tissues during protocols that elicit hemodynamic changes [8], and spectral measurements of the absolute optical properties of tissues [9,10]. When implemented to recover $\Delta \mu_{a}$ from a single type of optical data (e.g., only the phase of photon density waves (ϕ) in FD), the method is referred to as Dual-Slope (DS), which can been proposed in Time-Domain (TD) [11] and implemented in FD [12]. The basic “unit” for SC measurement features two sources and two detectors in a special geometrical arrangement. The four optodes identify four source–detector separations, and the prerequisite of a SC unit is that the two short and the two long source–detector separations must be equal. If this requirement is met (with some spatial constraints due to the dynamic range of detectors), one is free to choose between several spatial arrangements of the optodes. Typical ones are the linear (i.e. with the detectors sandwiched between the sources along a line), rectangular and trapezoidal (i.e., the optodes occupy the vertices of a rectangle and a trapezoid, respectively) [13]. Examples of these various arrangements can be found in the literature: linear [4-6,10-17]; rectangular [4,7,8,13,18-20]; and trapezoidal [13,20,21].

Since its inception the SC method has been also used with Continuous-Wave (CW) instrumentation. For example, Jenny et al. [18] developed a custom-made CW system with a probe having two concentric rectangular SC units and three wavelengths at each source location. The instrument was used for assessing the precision of StO_2_ measurements in vivo on a neonatal population. The same instrument was modified by Kleiser et al. [22] in a custom-made CW instrument (OxyPrem v. 1.3) featuring two collinear SC units, i.e., having two detectors and four source locations (four wavelengths per source location) sandwiched between the detectors in a linear array. The instrument was used to determine the accuracy of StO_2_ measurements on a blood-lipid phantom. Kleiser et al. [6] used the same instrument for StO_2_ precision assessment in a neonatal population. Chincarini et al. [21] used OxyPrem v. 1.4 featuring two SC units in a trapezoidal arrangement, and measured changes of oxy- and deoxyhemoglobin on sheep. Raoult et al. [23] used OxyPrem for measuring changes in oxy- and deoxyhemoglobin in dogs. Another commercial instrument, the Nonin EQUANOX, has used a probe with the SC arrangement to measure cerebral oxygenation in humans [14], but it is unclear if the SC method was implemented in the data analysis. Xu et al. [8] developed a custom-made CW system with eight linear SC arrangements (two wavelength per source location) combined in a hand-held probe for dynamic characterization of biological tissues. Scholkmann et al. [15] used OxyPrem to test the resilience of measurements towards induced motion artifacts. Perekatova et al. [17] used a custom-built CW ultra-broadband (460 nm to 1030 nm) featuring a SC linear unit and retrieved the effective attenuation coefficient ($\mu_{eff}$) at different wavelengths. The experimental setup was tested for resistance toward different instrumental perturbations. Wu et al. [7] developed a CW system with a probe comprising a SC unit in a rectangular arrangement (with two wavelengths per source location). The system was used for studying pulsatile cerebral blood flow together with a Diffuse Correlation Spectroscopy (DCS) system [19].

We note that when CW instrumentation is used for measuring StO_2_ or $\Delta \mu_{a}$, one must make assumptions about the values and wavelength dependence of the reduced scattering coefficient. This source of error and how it propagates to the target parameters has not been investigated. Also, there has not been a thorough investigation into the errors on the source–detector separations and how it propagates to the target parameters. This error was briefly addressed by Hueber et al. [4] for the linear arrangement by considering (a) errors in only one short distance; (b) errors in all the short distances; (c) errors in all short and long distances (by keeping the difference of the distances unchanged); (d) errors in all short and long distances by keeping the average distance unchanged. In this work, we consider a wider range of cases for the errors in the source–detector separation and their propagation for the quantification of absolute optical properties (i.e., $\mu_{eff}$ or $\mu_{a}$ and $\mu_{s}^{\prime }$), or absorption changes (i.e., $\Delta \mu_{a}$). We also consider four typical SC arrangements: linear, asymmetric-linear, trapezoidal, diagonal-rectangular.

## 2. Materials and Methods

### 2.1. Dual-Slope Arrangements

**Figure 1.**
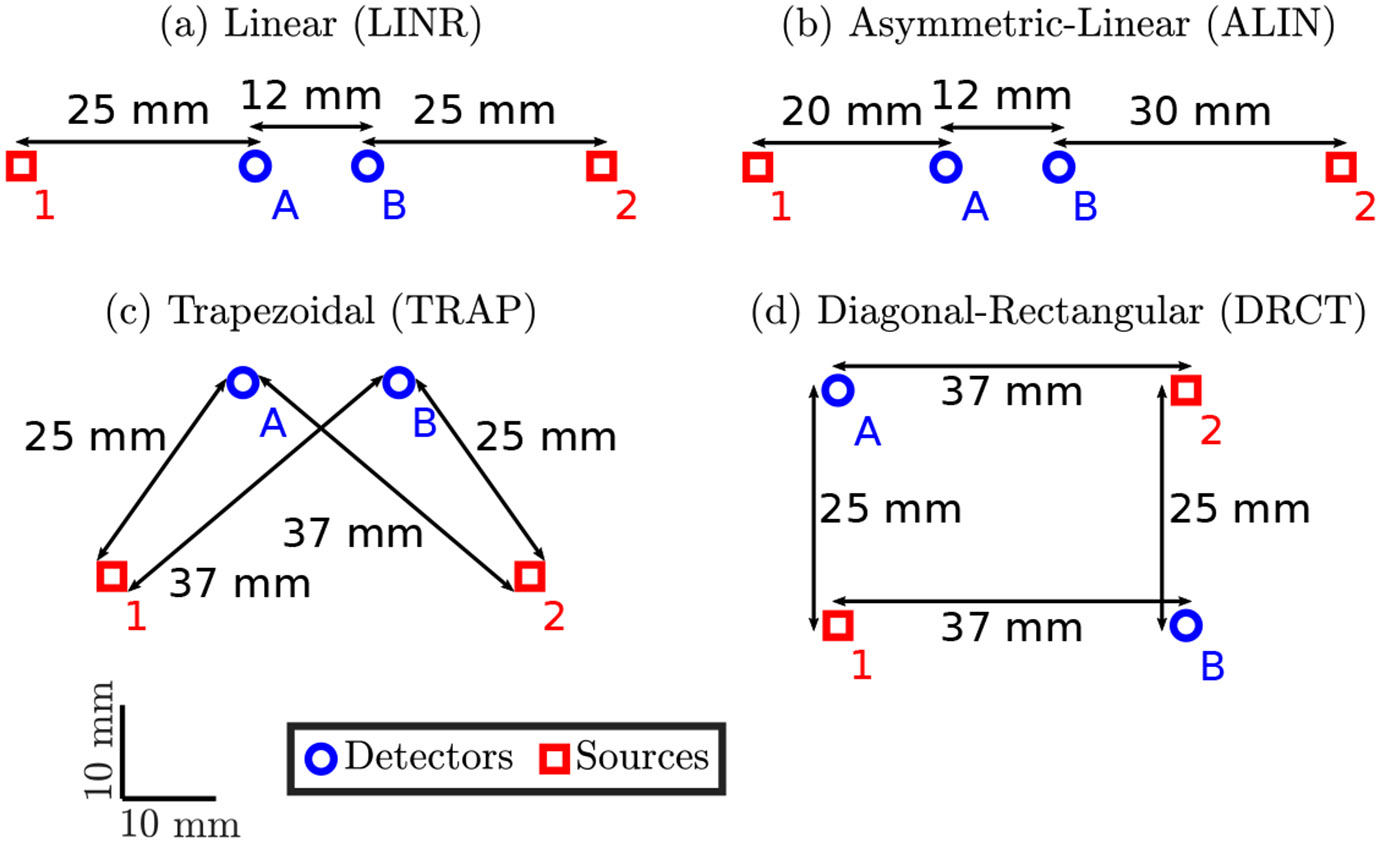
The four types of DS arrangement considered in this work. Sources are shown as red squares labeled with numbers and detectors are blue circles labeled with letters. Distances are measured from optode center to optode center. Drawn as a top-view such that the surface of the sample is represented as the plane of the page and the optodes are in contact with the sample. (**a**) The LINeaR (LINR) arrangement with source-detector distances (ρ’s) of [25, 25, 37, 37] mm. (**b**) The Asymmetric-LINear (ALIN) arrangement with ρ’s of [20, 30, 32, 42] mm. (**c**) The TRAPezoidal (TRAP) arrangement with ρ’s of [25, 25, 37, 37] mm. (**d**) The Diagonal-ReCTangular (DRCT) arrangement with ρ’s of [25, 25, 37, 37] mm.

In this work, four types of DS arrangement geometry will be considered (Figure 1). The first arrangement, LINeaR (LINR; Figure 1a), is the simplest and most common type of DS set. The next arrangement, Asymmetric-LINear (ALIN; Figure 1b), is a slight modification on LINR with the detectors shifted to the left. ALIN has not been implemented in as many experiments as the other sets, but has been shown to have a similar region of spatial sensitivity as LINR [13]. The last two sets, TRAPezoidal (TRAP; Figure 1c) and Diagonal-ReCTangular (DRCT; Figure 1c), are the two types of sets found in the modular hexagonal imaging arrangement proposed by our group [20]. TRAP is of particular interest since most DS imaging arrays are composed of mainly trapezoidal sets. All of these arrangements have the same mean source-detector distance (ρ) of 31 mm.

### 2.2. Analytical Forward Model

We generated forward FD NIRS data in the form of the complex Reflectance ($\tilde{R}$) for each source–detector pair in the arrangement being assessed (i.e., 1A, 1B, 2A, and 2B). These $\tilde{R}$ data were computed using an analytical diffusion model for a semi-infinite homogeneous medium [1]. The primary inputs to the forward model are $\mu_{a}$, $\mu_{s}^{\prime }$, and ρ, of which the former two were varied and the latter one was determined by the arrangement. Optical property ranges were chosen to encompass bulk tissue values recovered by FD NIRS on the human forehead [2]. The remaining model parameters were the modulation frequency ($f_{mod}$) and the index of refraction (n), which were fixed at 100 × 10^6^ Hz and 1.4, respectively. Importantly, when considering an optode position error, data were generated with ρ’s corresponding to the optode in the error position, not the nominal position.

We also considered changes in $\tilde{R}$ resulting from small changes in $\mu_{a}$ to test DS’s ability to recover $\Delta \mu_{a}’s$. These were computed by finding $\tilde{R}$ for the baseline optical properties and for the same parameters except for $\mu_{a}$, which was increased by 0.0001 mm^−1^ (i.e., true $\Delta \mu_{a}$ of 0.0001 mm^−1^).

### 2.3. Inverse Models

#### 2.3.1. Absolute Optical Properties

For a given simulation case, the forward model generated four values of $\tilde{R}$, one for each source–detector pair in the arrangement of interest. These four $\tilde{R}$s are input into the inverse models for recovering the $\mu_{a}$ and the $\mu_{s}^{\prime }$ to implement the SC method. The remaining parameters which must be assumed for the inverse models were the ρ’s, the $f_{mod}$, and the n. The ρ’s were either assigned the correct values (given the optode position considered in the forward model) or incorrect values. The cases where the ρ’s were assumed incorrectly correspond to cases where optodes were not in their nominal position to generate data with the forward model, but the nominal ρ’s were assumed in the inverse model. This represents a possible real-life scenario where the optode arrangement was affected by position errors, but the data were analyzed assuming the nominal ρ’s. The remaining two parameters, the $f_{mod}$ and the n, were assumed to be known in all cases.

In this work, we have tested two inverse models based on the SC $\tilde{R}$ data to recover the $\mu_{a}$ and the $\mu_{s}^{\prime }$. We will refer to the first method as the “slopes” method since it is based on the slopes, computed with SC/DS methods, of the linearized Intensity ($\text{ln}(\rho^{2}I)$) and the phase of photon density waves (ϕ) with respect to ρ [2]. The second method we will refer to as the “iterative” method since it is based on iteratively solving the forward model for $\tilde{R}$ as a function of ρ [16], where $\tilde{R}=Ie^{i\phi }$. The “slopes” method solves the following equation:

(1) $$\text{ln}\left[\rho^{2}\tilde{R}\right]=-{\tilde{\mu }}_{eff}\rho$$

where the complex effective attenuation coefficient (${\tilde{\mu }}_{eff}$) is defined as:

(2) $${\tilde{\mu }}_{eff}=\sqrt{3(\mu_{s}^{\prime }+\mu_{a})(\mu_{a}-2\pi f_{mod}i∕v)}$$

where v is the speed of light in the medium. The “iterative” method, on the other-hand, iteratively solves this expression:

(3) $$\text{ln}\left[\tilde{f}(\rho ,\mu_{a},\mu_{s}^{\prime })\tilde{R}\right]=-{\tilde{\mu }}_{eff}\sqrt{\rho^{2}+1∕\mu_{s}^{\prime 2}}$$

by updating the $\mu_{a}$ and the $\mu_{s}^{\prime }$ every iteration. Details on the iterative method and the complex function $\tilde{f}(\rho ,\mu_{a},\mu_{s}^{\prime })$ can be found in Reference [16]’s Equation (4). The slope method is based on stricter assumptions than the iterative method but is less computationally intense and more stable against artifacts in noisy data.

#### 2.3.2. Changes in Absorption

The forward model considered changes in the $\mu_{a}$ by generating $\tilde{R}$s for both the baseline optical properties and perturbed optical properties for each of the four source–detector pairs in the DS arrangement of interest. From these baseline and perturbed $\tilde{R}$s, the changes in the DS of optical absorbance ($\text{ln}(I_{0}∕I)$) (i.e., $\text{ln}(|\tilde{R}|)$) or ϕ (i.e., $∠\tilde{R}$) were calculated and converted to $\Delta \mu_{a}$ using previously reported methods [12]. It is worth noting that these methods require an assumption of $\mu_{a}$ and $\mu_{s}^{\prime }$ to calculate the general complex average total optical path-lengths ($\langle \tilde{L}\rangle s$). The values of $\mu_{a}$ and $\mu_{s}^{\prime }$ found using the iterative method on the forward data were used in this case, meaning errors in the recovered $\mu_{a}$ and $\mu_{s}^{\prime }$ may propagate through $\langle \tilde{L}\rangle s$ to an error on the recovered $\Delta \mu_{a}$. The inverse model for absorption changes also requires an assumption of the ρ’s. These ρ’s were assumed in the same way as described for the absolute optical property inverse model, with the nominal ρ’s always used in the inverse problem even when an error is imposed on the optode positions to generate the data with the forward model.

### 2.4. Variable Definitions

The results in this work focus on errors in recovered parameters as a result of incorrect assumed values of ρ due to geometrical optode position errors. Therefore, we must be careful to use a notation which differentiates true parameters from parameters recovered either with correct or incorrect assumptions. Accordingly, variables with (√ρ) in superscript indicate values recovered by the inverse model assuming the correct ρ’s, while variables with (×ρ) in superscript indicate values recovered assuming the incorrect ρ’s. Variables without either (√ρ) or (×ρ) in superscript indicate the true values used in the forward model to generate simulated data. For example, $\mu_{a}$ indicates the true absorption coefficient used in the forward model, while $\mu_{a}^{\left(\surd \rho \right)}$ or $\mu_{a}^{\left(\times \rho \right)}$ indicate the absorption coefficient recovered by the inverse model, assuming the correct or incorrect ρ’s, respectively.

We also aim to assess to what extent an error in an optode’s position affects the values recovered in general. To quantify this we define the root-mean-square-error in either the $\mu_{a}$ or the $\mu_{s}^{\prime }$ by varying the optode positions in a 2 mm diameter circle which orbits their nominal position ($\sigma_{\mu_{a}}^{\emptyset 2\;\text{mm}}$ or $\sigma_{\mu_{s}^{\prime }}^{\emptyset 2\;\text{mm}}$, respectively) as follows:

(4) $$\sigma_{\mu_{a}}^{\emptyset 2\;\text{mm}}=\sqrt{\frac{\sum_{i=1}^{n^{\emptyset 2\;\text{mm}}}{\left(\mu_{a,i}^{\left(\times \rho \right)}-\mu_{a}^{\left(\surd \rho \right)}\right)}^{2}}{n^{\emptyset 2\;\text{mm}}}}$$

(5) $$\sigma_{\mu_{s}^{\prime }}^{\emptyset 2\;\text{mm}}=\sqrt{\frac{\sum_{i=1}^{n^{\emptyset 2\;\text{mm}}}{\left(\mu_{s^{\prime },i}^{\prime (\times \rho )}-\mu_{s^{\prime }}^{\prime (\surd \rho )}\right)}^{2}}{n^{\emptyset 2\;\text{mm}}}}$$

where $\mu_{a}^{\left(\surd \rho \right)}$ and $\mu_{s^{\prime }}^{\prime (\surd \rho )}$ are the absorption coefficient and the reduced scattering coefficient recovered by the inverse model with the optode in its nominal position and assuming the nominal ρ’s. However, $\mu_{a}^{\left(\times \rho \right)}$ and $\mu_{s^{\prime }}^{\prime (\times \rho )}$ are the absorption coefficient and the reduced scattering coefficient recovered by the inverse model with the optode *not in its nominal position but assuming the nominal* ρ’s. To realize this, the optode’s (e.g., source 1’s) position is varied through $n^{\emptyset 2\;\text{mm}}$ points in a 2 mm diameter circle surrounding its nominal position (e.g., a circle orbiting source 1’s nominal position with a 1 mm radius) and computing the forward then inverse models for each point. To assess the influence of a position error in multiple optodes (e.g., source 1 and detector A), each optode considers $\sqrt[{m}]{n^{\emptyset 2\;\text{mm}}}$ positions (e.g., $\sqrt{n^{\emptyset 2\;\text{mm}}}$) in a circle around its nominal position, where m is the number of optodes considered (e.g., 2). These positions are co-varied for each optode resulting in $n^{\emptyset 2\;\text{mm}}$ values of $\mu_{a}^{\left(\times \rho \right)}$ and $\mu_{s^{\prime }}^{\prime (\times \rho )}$, which may be used to compute $\sigma_{\mu_{a}}^{\emptyset 2\;\text{mm}}$ or $\sigma_{\mu_{s}^{\prime }}^{\emptyset 2\;\text{mm}}$ with Equations (4) and (5). It should be noted that this definition quantifies deviation from the recovered nominal value instead of deviation from the true value to remove any effect of systematic error in the inversion.

## 3. Results

### 3.1. Self-Calibrated Recovery of Absolute Optical Properties

Before diving into the errors introduced by recovery method or optode position, we present the expected errors in $\mu_{a}$ and $\mu_{s}^{\prime }$ from other common experimental considerations to form a point of comparison. First, the recovery methods typically assume point-like optical sources and detectors. However, in reality optode areas are finite. Therefore, to understand the error introduced by this assumption, we have simulated a situation with source radii of 300 μm and detector radii of 1.5 mm. In the case of a $\mu_{a}$ of 0.01 mm^−1^, a $\mu_{s}^{\prime }$ of 1 mm^−1^, and ρ’s of [25, 25, 37, 37] mm, assuming these finite optode sizes creates an additional error in $\mu_{a}$ and $\mu_{s}^{\prime }$ of 0.1% and 0.2%, respectively, when using the iterative recovery method. Since this consideration of optode area creates little additional error, we modeled optodes as point-like for the remainder of this work. However, we do note that if the optode sizes are known, then this consideration can be included in the inverse model, which may be relevant at shorter source–detector separations of the order of 10 mm or less. Second, in reality, random instrumental noise propagates to a random error in the recovered $\mu_{a}$ and $\mu_{s}^{\prime }$. Considering typical instrumental noise values of 0.1% for the Intensity (I) and 0.1° for the ϕ results in random error in $\mu_{a}$ and $\mu_{s}^{\prime }$ of 0.6% and 0.5%, respectively, using the same parameters as the finite optode case above. Since instrumental noise is also expected to propagate to small errors in optical properties, this additional consideration is also neglected for the remainder of the manuscript.

#### 3.1.1. Error from the Choice of Optical Property Recovery Method

We have compared the accuracy of two methods to recover the $\mu_{a}$ and the $\mu_{s}^{\prime }$ from SC FD NIRS data. These two methods are the slopes method [2] and the iterative method [16], where the slope method is simpler but contains stronger assumptions in its derivation. Table 1 shows the accuracy of $\mu_{a}^{\left(\surd \rho \right)}$ and $\mu_{s^{\prime }}^{\prime (\surd \rho )}$ recovered using each method for a range of true values of $\mu_{a}$ and $\mu_{s}^{\prime }$. We conducted this exercise for two sets of ρ’s. The first set, ρ=[25,25,37,37]mm, corresponds to the LINR, TRAP, and DRCT sets (Figure 1a,c,d). The second set, ρ=[20,30,32,42]mm, corresponds to the ALIN set (Figure 1b).

Overall, the results in Table 1 show that the slopes method is less accurate than the iterative method. To summarize, averaging the error in $\mu_{a}^{\left(\surd \rho \right)}$ for all optical properties and ρ sets yields 6% and 0.01% for the slopes and iterative methods, respectively. Similarly, averaging the error in $\mu_{s^{\prime }}^{\prime (\surd \rho )}$ for all optical properties and ρ sets yields −6 % and −1 % for the slopes and iterative methods, respectively. Both methods have a positive bias for $\mu_{a}^{\left(\surd \rho \right)}$ and a negative bias for $\mu_{s^{\prime }}^{\prime (\surd \rho )}$. In either case, the accuracy of the method depends strongly on the optical properties and only depends weakly on the set of ρ’s, so we will focus on interpreting the results for the ρ=[25,25,37,37]mm set of ρ’s here. The accuracy of the $\mu_{a}^{\left(\surd \rho \right)}$ recovered by the slopes method depends heavily on the true $\mu_{a}$, with worse accuracies occurring at low $\mu_{a}$s. Meanwhile, the accuracy of the $\mu_{a}^{\left(\surd \rho \right)}$ recovered by the iterative method more strongly depends on the true $\mu_{s^{\prime }}^{\prime }$, and only weakly depends on the true $\mu_{a}$. For both the slopes and iterative methods, the accuracy of $\mu_{s^{\prime }}^{\prime (\surd \rho )}$ depends on the true value of $\mu_{s^{\prime }}^{\prime }$, with lower true $\mu_{s}^{\prime }s$ corresponding to worse accuracy. The purpose of these results is to indicate how accurate these methods are even in the case where the ρ’s are known. From these results, we decided to focus mainly on the iterative method and only show select results for the slopes method due to the iterative method’s substantially better accuracy.

#### 3.1.2. Error from Optode Position Displacement

##### Single-Optode Displacement

###### Linear (LINR) Arrangement

**Figure 2.**
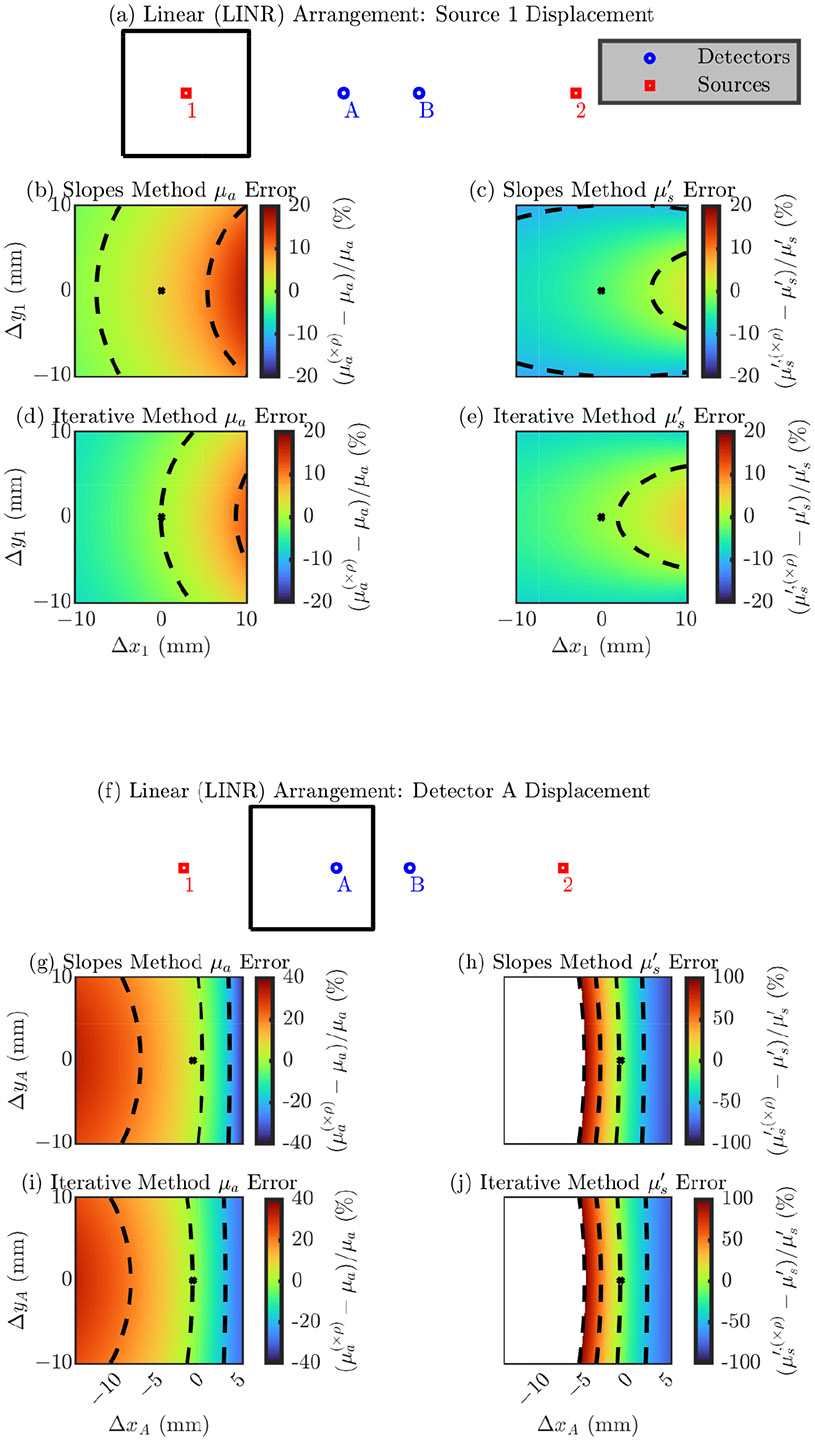
Error in the accuracy of the “slopes” or “iterative” optical recovery method for the LINeaR (LINR) arrangement. In the color maps the black dashed iso-lines represent the color bar tick-mark values and the nominal optode position is shown as a black asterisk. (**a**–**e**) Errors from displacement of source 1 (equivalent to source 2). (**f**–**j**) Errors from displacement of detector A (equivalent to detector B). (**b**,**c**,**g**,**h**) Results using the “slopes” [2] recovery method. (**d**,**e**,**i**,**j**) Results using the “iterative” [16] recovery method. (**b**,**d**,**g**,**i**) Error in the absorption coefficient ($\mu_{a}$). (**c**,**e**,**h**,**j**) Error in the reduced scattering coefficient ($\mu_{s}^{\prime }$). Symbols: Displacement of optode from the nominal position ([Δx, Δy]), optical property recovered with the optode displaced from the nominal position ((×ρ) in superscript).

We begin investigating optode position errors by exploring errors in a single optode’s position in the LINR arrangement. Due to the symmetry in this arrangement, displacing either source 1 or source 2 will result in the same error. Similarity, displacement in either detector A or detector B will also result in the same error. Therefore, we only show results for displacements of source 1 or detector A. Figure 2 shows the error in the recovery optical properties ($\mu_{a}^{\left(\times \rho \right)}$ and $\mu_{s^{\prime }}^{\prime (\times \rho )}$) relative to the true optical properties ($\mu_{a}$ and $\mu_{s}^{\prime }$) due to displacing the optode from its nominal position in a 20 mm × 20 mm square. The recovered optical properties always assume the nominal optode position even if the optode is not in that position, thus the use of the (×ρ) superscript.

To interpret the results in Figure 2, we will focus on the direction of maximum change in the error and the range of the error from displacement in the 20 mm × 20 mm square. Concentrating on the subplots for source 1 (Figure 2a-e), we find four color maps for each combination of the two recovery methods “slopes” or “iterative” (Figure 2b,c or d,e, respectively) and two optical properties $\mu_{a}$ or $\mu_{s}^{\prime }$ (Figure 2b,d or c,e, respectively). All four of these subplots show that larger errors occur if the optode is displaced away or toward the detectors (along the linear line of the LINR arrangement). In each case, the range of the error is about 10%. Now focusing on errors from displacement of detector A (Figure 2f-j), we see again that movement along the linear line of the arrangement creates large changes in the error. However, the magnitude of the error from displacing detector A is much larger than source 1. Displacement of detector A in the 20 mm × 20 mm square results in a error range of about 30% for $\mu_{a}$ and >100 % for $\mu_{s}^{\prime }$ (for both the slopes and iterative recovery methods). Overall, Figure 2 shows that displacement of optodes along the line of the LINR arrangement creates the largest error and the position of the detectors is much more critical than the position of the sources, but the two recovery methods do not have a significantly different dependence on optode position error.

Table 2 represents the magnitude of the error in the recovered optical properties from a 1 mm displacement in any direction. This is done using the metrics $\sigma_{\mu_{a}}^{\emptyset 2\;\text{mm}}\;∕\;\mu_{a}^{\left(\surd \rho \right)}$ or $\sigma_{\mu_{s}^{\prime }}^{\emptyset 2\;\text{mm}}\;∕\;\mu_{s^{\prime }}^{\prime (\surd \rho )}$ (Equation (4) or Equation (5)), which represent the average error resulting from displacing an optode 1 mm in any direction from its nominal position relative to the value the method would recover with the optode in the nominal position ($\mu_{a}^{\left(\surd \rho \right)}$ or $\mu_{s^{\prime }}^{\prime (\surd \rho )}$). These values are important because they represent expected errors that could result from probe construction, since it is reasonable that optode positions in a real probe may be incorrect on the order of 1 mm. Therefore, these errors can be thought of as expected systematic errors from the practical limitations of building a NIRS probe, for a given arrangement, optical property, and recovery method. Specifically for the LINR arrangement, we see that an error in the detector position propagates to a worse error in optical properties in all cases. Additionally, the effect of a 1 mm displacement does not seem significantly different between the slopes and iterative methods. Aside from that, the remaining conclusions are somewhat expected from the limitations of diffusion theory. That is, overall larger errors in $\mu_{s}^{\prime }$ than $\mu_{a}$ (specifically for the case of detector displacement) and worse errors at low true values of $\mu_{a}$ and $\mu_{s}^{\prime }$. For overall guidance, from a 1 mm optode position error in any direction, we can expect about a 0.7% or 3% error, for source or detector displacement, respectively, in $\mu_{a}$ and about a 0.3% or 10% error, for source or detector displacement, respectively, in $\mu_{s}^{\prime }$ (using the iterative recovery method) for the LINR arrangement.

###### Asymmetric-Linear (ALIN) Arrangement

Second, we explore errors in a single optode’s position in the ALIN arrangement. Due to the lack of symmetry in this arrangement, displacement of each optode must be looked at individually.

**Figure 3.**
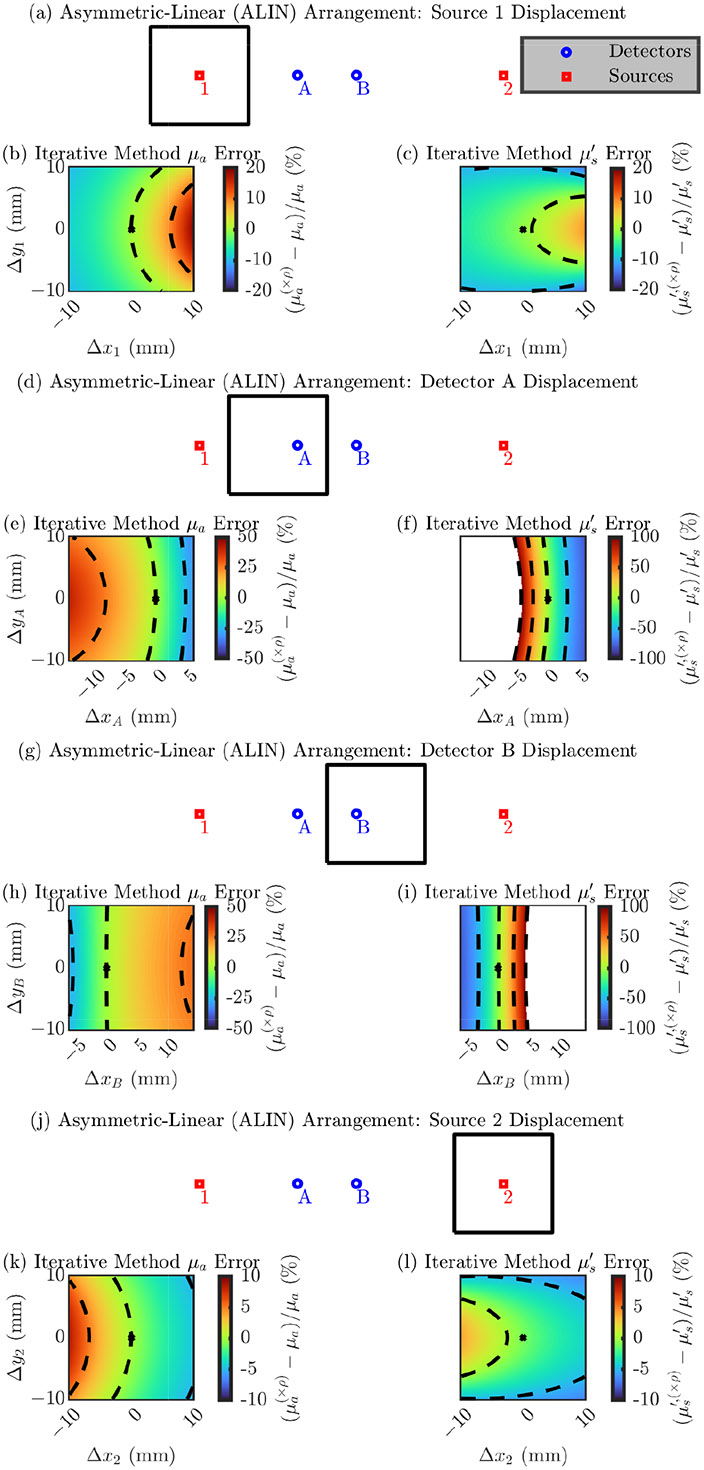
Error in the accuracy of the iterative optical recovery method for the Asymmetric-LINear (ALIN) arrangement. In the color maps the black dashed iso-lines represent the color bar tick-mark values and the nominal optode position is shown as a black asterisk. (**a**–**c**) Errors from displacement of source 1. (**d**–**f**) Errors from displacement of detector A. (**g**–**i**) Errors from displacement of detector B. (**j**–**l**) Errors from displacement of source 2. (**b**,**e**,**h**,**k**) Error in the absorption coefficient ($\mu_{a}$). (**c**,**f**,**i**,**l**) Error in the reduced scattering coefficient ($\mu_{s}^{\prime }$). Symbols: Displacement of optode from the nominal position ([Δx, Δy]), optical property recovered with the optode displaced from the nominal position ((×ρ) in superscript).

Interpreting the results in Figure 3, we again focus on the direction of maximum change in the error and the range of the error in the simulated square. Similar to the LINR arrangement in Figure 2, all of the subplots show that larger errors occur if the optode is displaced away or toward the detectors (along the linear line of the ALIN arrangement). Looking at the range of the error, we see that the position of the detectors is most critical with an error range of about 60% for $\mu_{a}$ and >100 % for $\mu_{s}^{\prime }$. However, the position of source 1 is slightly more critical than the position of source 2. Source 1 shows an error range of about 20% for $\mu_{a}$ and $\mu_{s^{\prime }}^{\prime }$, while source 2 shows a range of about 10%. Overall, Figure 3 shows that displacement of optodes along the line of the ALIN arrangement creates the largest error and the position of the detectors is much more critical than the position of the sources, same as the LINR arrangement (Figure 2).

Table 3 presents the magnitude of the error in the recovered optical properties from a 1 mm displacement in any direction for the ALIN arrangement (Equation (4) or Equation (5)). Similar to the LINR arrangement, we see for the ALIN arrangement that errors in the detector position propagate to a worse error in optical properties in all cases. To give overall guidance, from a 1 mm optode position error in any direction, we can expect about a 0.7% or 3% error, for source or detector displacement, respectively, in $\mu_{a}$ and about a 0.4% or 10% error, for source or detector displacement, respectively, in $\mu_{s}^{\prime }$ (using the iterative recovery method) for the ALIN arrangement.

###### Trapezoidal (TRAP) Arrangement

Next, we explore errors in a single optode’s position in the TRAP arrangement. The symmetry of the TRAP arrangement lets us focus on only source 1 (since it is equivalent to source 2) and detector A (since it is equivalent to detector B).

**Figure 4.**
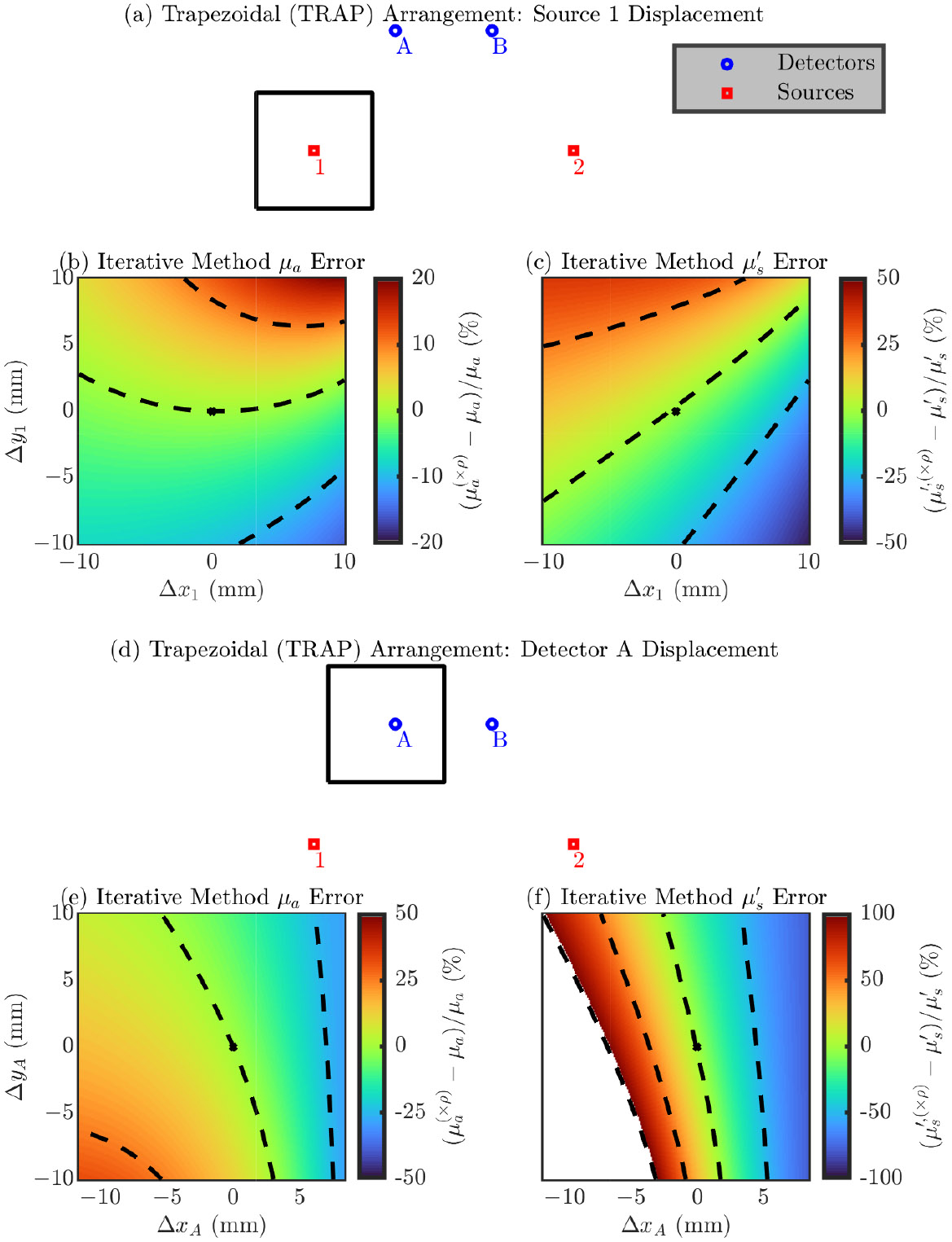
Error in the accuracy of the iterative optical recovery method for the TRAPezoidal (TRAP) arrangement. In the color maps the black dashed iso-lines represent the color bar tick-mark values and the nominal optode position is shown as a black asterisk. (**a**–**c**) Errors from displacement of source 1 (equivalent to source 2). (**d**–**f**) Errors from displacement of detector A (equivalent to detector B). (**b**,**e**) Error in the absorption coefficient ($\mu_{a}$). (**c**,**f**) Error in the reduced scattering coefficient ($\mu_{s}^{\prime }$). Symbols: Displacement of optode from the nominal position ([Δx, Δy]), optical property recovered with the optode displaced from the nominal position ((×ρ) in superscript).

Focusing on the results in Figure 4, we again emphasize the direction of maximum change in the error and the range of the error in the simulated square. In this arrangement the directions which cause a maximum change in the error are not as trivial as they were in the LINR and ALIN arrangements. Looking at the range of the error, we again see that the position of the detectors is most critical with an error range for detector A of about 60% for $\mu_{a}$ and >100 % for $\mu_{s}^{\prime }$. Instead, source 1 shows an error range of about 30% for $\mu_{a}$ and about 70% for $\mu_{s}^{\prime }$. Overall, Figure 4 shows that the direction of the optode displacement which causes the maximum error is not as trivial as it is with the LINR or ALIN arrangements. We will discuss a way to use knowledge of these directions in Section Design of Optode Support Structure.

Table 4 shows the magnitude of the error in the recovered optical properties from a 1 mm displacement in any direction for the TRAP arrangement (Equation (4) or Equation (5)). Similar to the LINR and ALIN arrangement, we see for the TRAP arrangement that errors in the detector position propagate to a worse error in optical properties in all cases; however, the difference in the dependence on source or detector is not as strong with the TRAP arrangement. To give overall guidance, from a 1 mm optode position error in any direction, we can expect about a 0.9% or 2% error, for source or detector displacement, respectively, in $\mu_{a}$ and about a 2% or 9% error, for source or detector displacement, respectively, in $\mu_{s}^{\prime }$ (using the iterative recovery method) for the TRAP arrangement.

###### Diagonal-Rectangular (DRCT) Arrangement

Finally, we explore errors in a single optode’s position in the DRCT arrangement. The symmetry of the DRCT arrangement lets us focus on only source 1 (since it is equivalent to source 2, detector A, and detector B).

**Figure 5.**
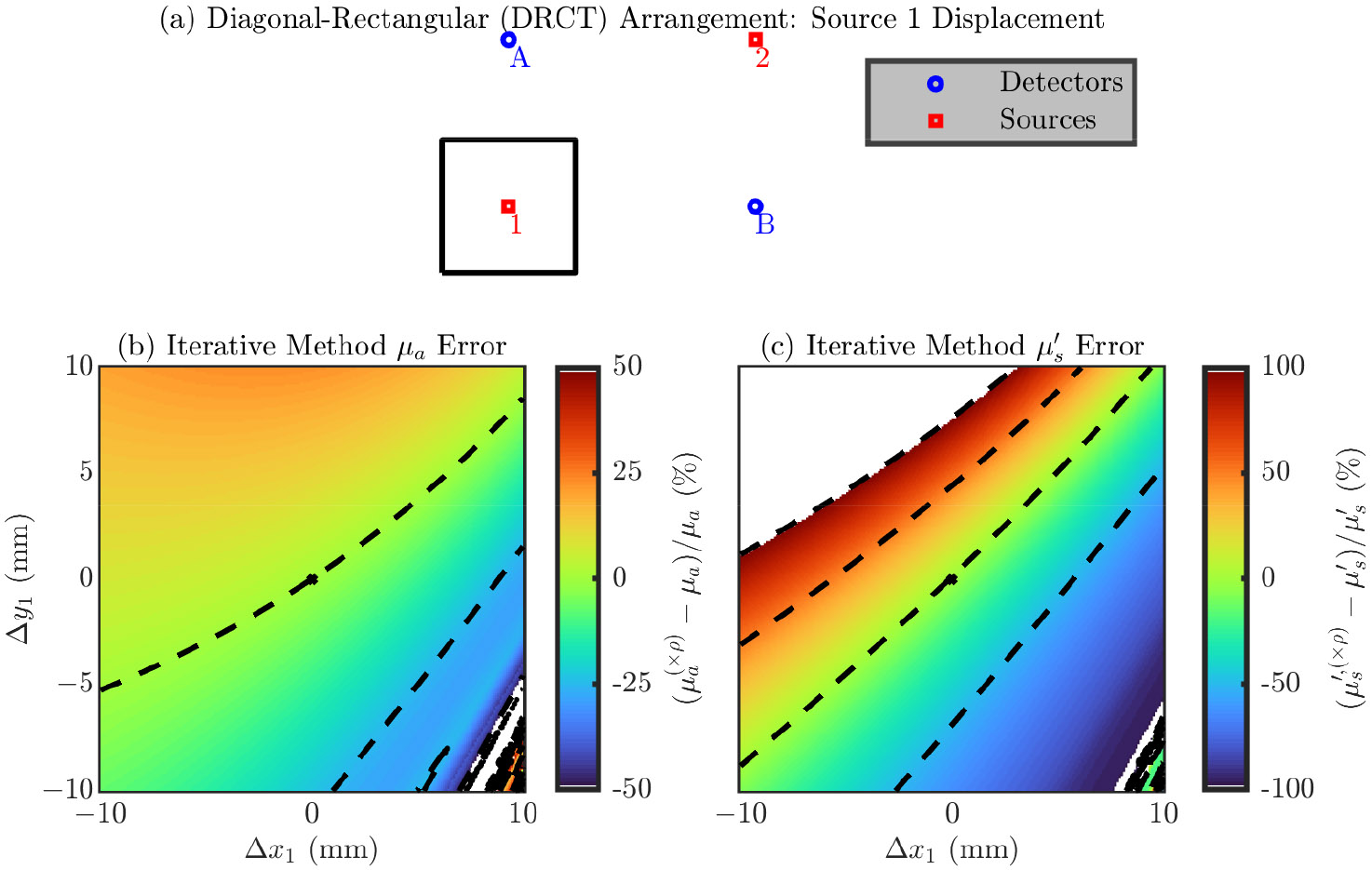
Error in the accuracy of the iterative optical recovery method for the Diagonal-ReCTangular (DRCT) arrangement. In the color maps the black dashed iso-lines represent the color bar tick-mark values and the nominal optode position is shown as a black asterisk. (**a**–**c**) Errors from displacement of source 1 (equivalent to source 2, detector A, and detector B). (**b**) Error in the absorption coefficient (μ). (**c**) Error in the reduced scattering coefficient ($\mu_{s}^{\prime }$). Symbols: Displacement of optode from the nominal position ([Δx, Δy]), optical property recovered with the optode displaced from the nominal position ((×ρ) in superscript).

Figure 5 shows the results for the DRCT arrangement. Similar to the TRAP arrangement, the directions which cause a maximum change in the error are not as trivial as they were in the LINR and ALIN arrangements. However, roughly speaking, it seems that displacement of an optode towards or away from the center causes little error (i.e., movement orthogonal to that creates a large change in the error). Since, for this arrangement, displacement of any optode is equivalent, there is no error dependence preference between sources and detectors (i.e., their positions are equally important). Looking at the range of the error, we see an error range of about >100% for $\mu_{a}$ and >100% for $\mu_{s}^{\prime }$ from moving with the 20 mm × 20 mm square. However, the large (i.e., >100%) errors occur for $\mu_{a}$ only in the lower right corner of the square; ignoring that region, the $\mu_{a}$ error range is about 50%.

Table 5 shows the magnitude of the error in the recovered optical properties from a 1 mm displacement in any direction for the DRCT arrangement (Equation (4) or Equation (5)). In this case there is no dependence on optode, since displacement of any optode is equivalent. To give overall guidance, from a 1 mm optode position error in any direction, we can expect about a 2% in $\mu_{a}$ and about a 9% in $\mu_{s}^{\prime }$ (using the iterative recovery method) for the DRCT arrangement.

##### Multi-Optode Displacement

Table 6 shows the average errors which may be expected if multiple optodes are displaced by 1 mm in any direction assuming 1.0 mm^−1^ for $\mu_{s}^{\prime }$ and 0.01 mm^−1^ for $\mu_{a}$. Certain combinations were omitted due to symmetry as follows (the “≡” symbol is used to indicate that an optode combination is equivalent): for LINR and TRAP 1A ≡ 2B, 1B ≡ 2A, 1AB ≡ 2AB, and 12A ≡ 12B; for ALIN no combination is omitted; and for DRCT 1A ≡ 2B, 1B ≡ 2A, 12 ≡ AB, and 1AB ≡ 2AB ≡ 12A ≡ 12B. The errors in this table are, for the most part, no more than twice the worst-case error from a single optode displacement. Furthermore, moving from two optodes to three to four does not significantly change the magnitude of the error, with more dependence on if a critical optode is displaced (e.g., the detectors in the LINR arrangement). For example, in the LINR case, the lowest error occurs by displacing 12, which is the only case that does not include a detector displacement. Furthermore, in this case 12A shows an error more compatible to the 1A and 1B cases then the 1AB case; therefore, the main influence in the magnitude of the error appears to be how many detectors are displaced (i.e., more generally, how many critical optodes are displaced). Overall, these results point to the TRAP arrangement being slightly more robust against optode displacement than the other arrangements, possibly because it de-emphasizes the criticality of the detector positions. This can be seen in the case where all four optodes are displaced (12AB), which results in TRAP average errors of about 3% and 14%, for $\mu_{a}$ and $\mu_{s}^{\prime }$, respectively, while this average error is 4% and 19%, for $\mu_{a}$ and $\mu_{s}^{\prime }$, respectively, for the LINR, ALIN, and DRCT arrangements.

### 3.2. Dual-Slope Recovery of Relative Changes in the Absorption coefficient ($\mu_{a}$)

**Figure 6.**
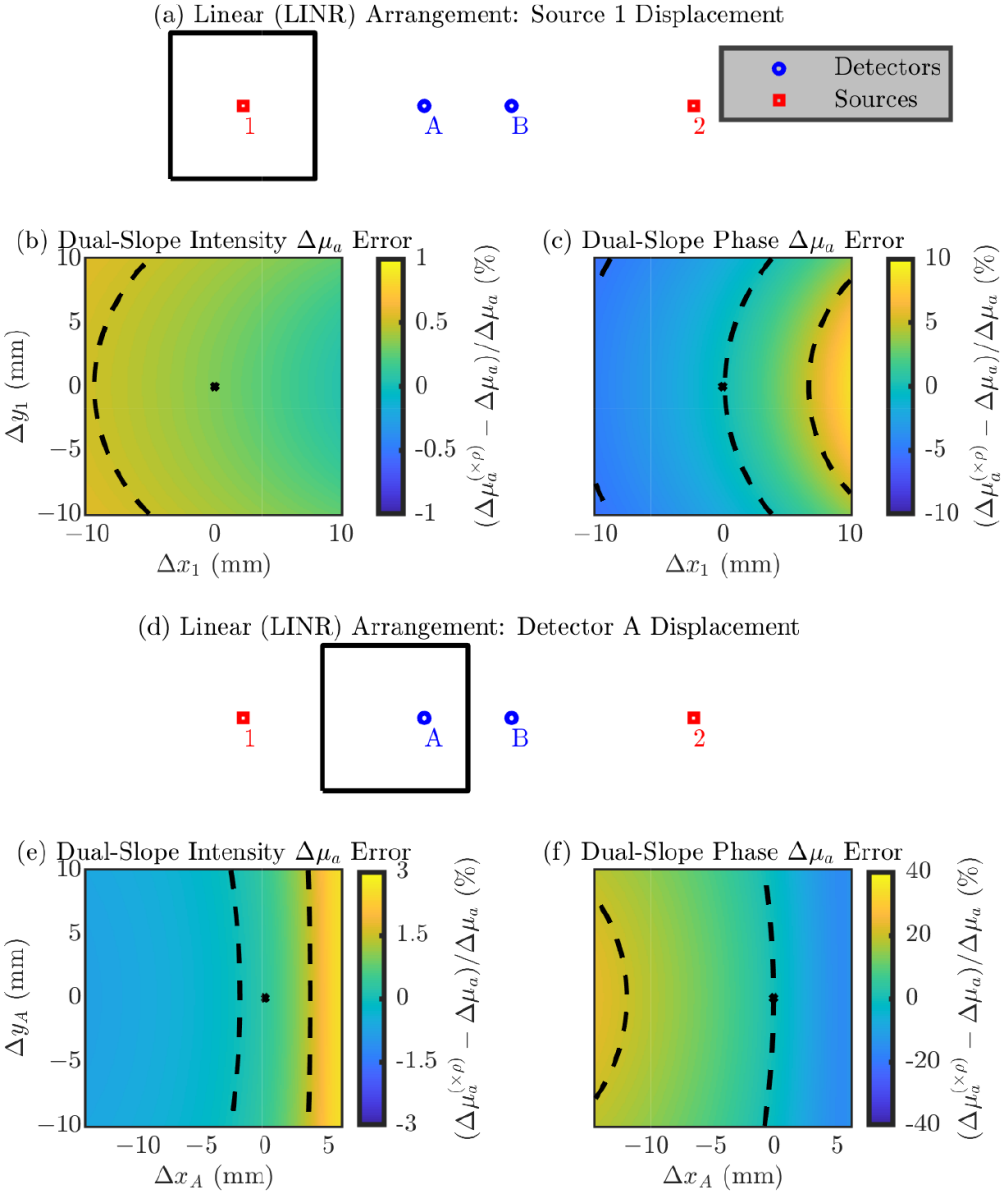
Error in the accuracy of recovering absorption coefficient change ($\Delta \mu_{a}$) for the LINeaR (LINR) arrangement. In the color maps the black dashed iso-lines represent the color bar tick-mark values and the nominal optode position is shown as a black asterisk. (**a**–**c**) Errors from displacement of source 1 (equivalent to source 2). (**d**–**f**) Errors from displacement of detector A (equivalent to detector B). (**b**,**e**) Error in the $\Delta \mu_{a}$ recovered by Dual-Slope (DS) Intensity (I). (**c**,**f**) Error in the $\Delta \mu_{a}$ recovered by DS phase of photon density waves (ϕ). Symbols: Displacement of optode from the nominal position ([Δx, Δy]), optical property recovered with the optode displaced from the nominal position ((×ρ) in superscript).

**Figure 7.**
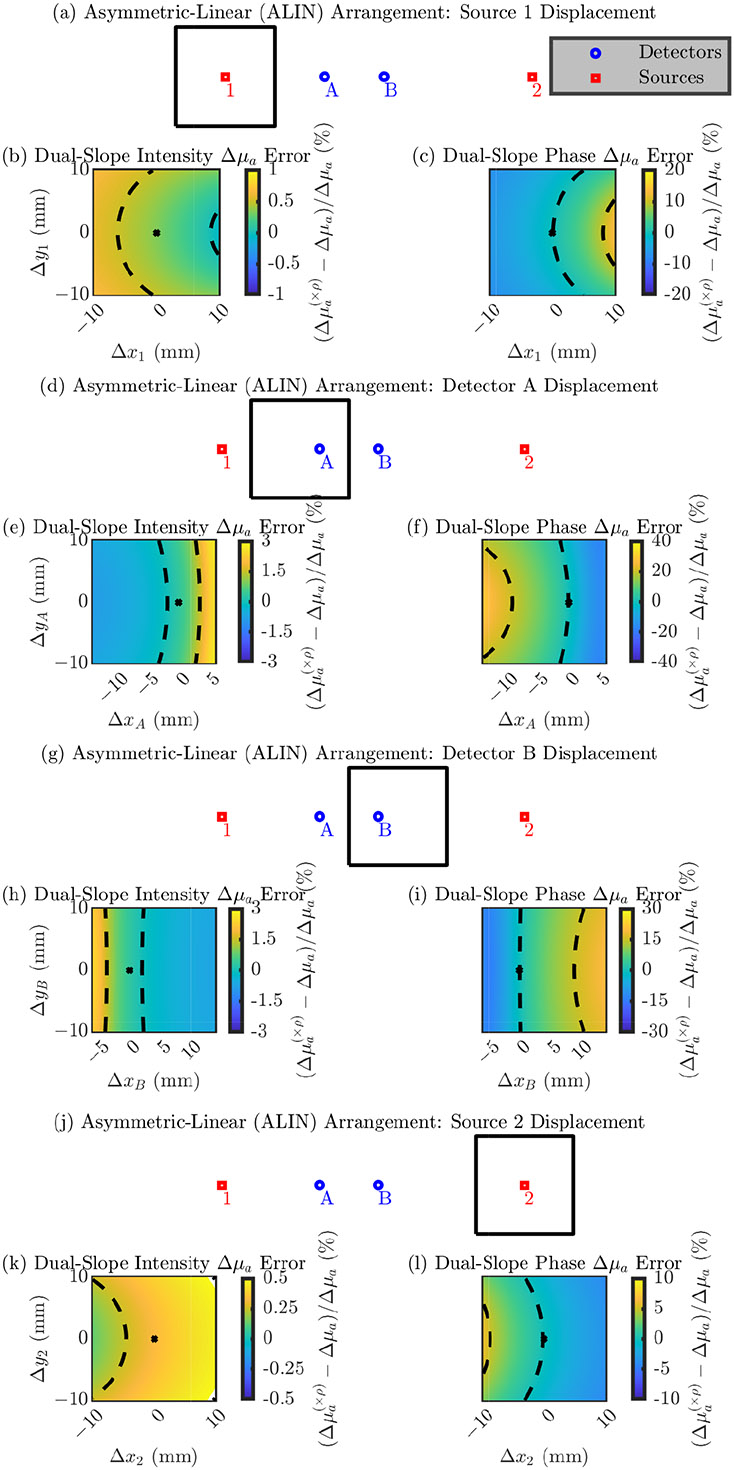
Error in the accuracy of recovering absorption coefficient change ($\Delta \mu_{a}$) for the Asymmetric-LINear (ALIN) arrangement. In the color maps the black dashed iso-lines represent the color bar tick-mark values and the nominal optode position is shown as a black asterisk. (**a**–**c**) Errors from displacement of source 1. (**d**–**f**) Errors from displacement of detector A. (**g**–**i**) Errors from displacement of detector B. (**j**–**l**) Errors from displacement of source 2. (**b**,**e**,**h**,**k**) Error in the $\Delta \mu_{a}$ recovered by Dual-Slope (DS) Intensity (I). (**c**,**f**,**i**,**l**) Error in the $\Delta \mu_{a}$ recovered by DS phase of photon density waves (ϕ). Symbols: Displacement of optode from the nominal position ([Δx, Δy]), optical property recovered with the optode displaced from the nominal position ((×ρ) in superscript).

**Figure 8.**
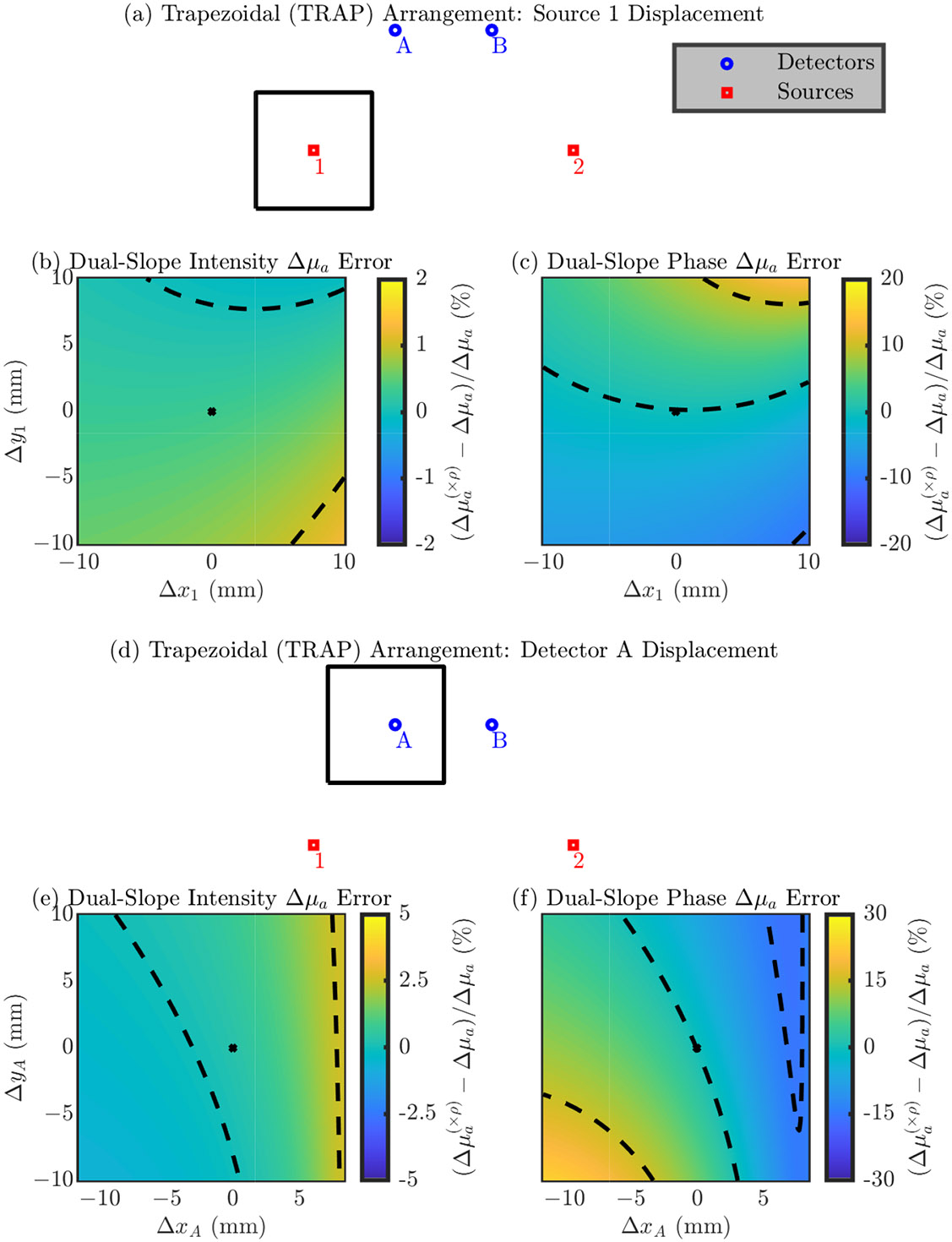
Errorin the accuracy of recovering absorption coefficient change ($\Delta \mu_{a}$) for the TRAPezoidal (TRAP) arrangement. In the color maps the black dashed iso-lines represent the color bar tick-mark values and the nominal optode position is shown as a black asterisk. (**a**–**c**) Errors from displacement of source 1 (equivalent to source 2). (**d**–**f**) Errors from displacement of detector A (equivalent to detector B). (**b**,**e**) Error in the $\Delta \mu_{a}$ recovered by Dual-Slope (DS) Intensity (I). (**c**,**f**) Error in the $\Delta \mu_{a}$ recovered by DS phase of photon density waves (ϕ). Symbols: Displacement of optode from the nominal position ([Δx, Δy]), optical property recovered with the optode displaced from the nominal position ((×ρ) in superscript).

**Figure 9.**
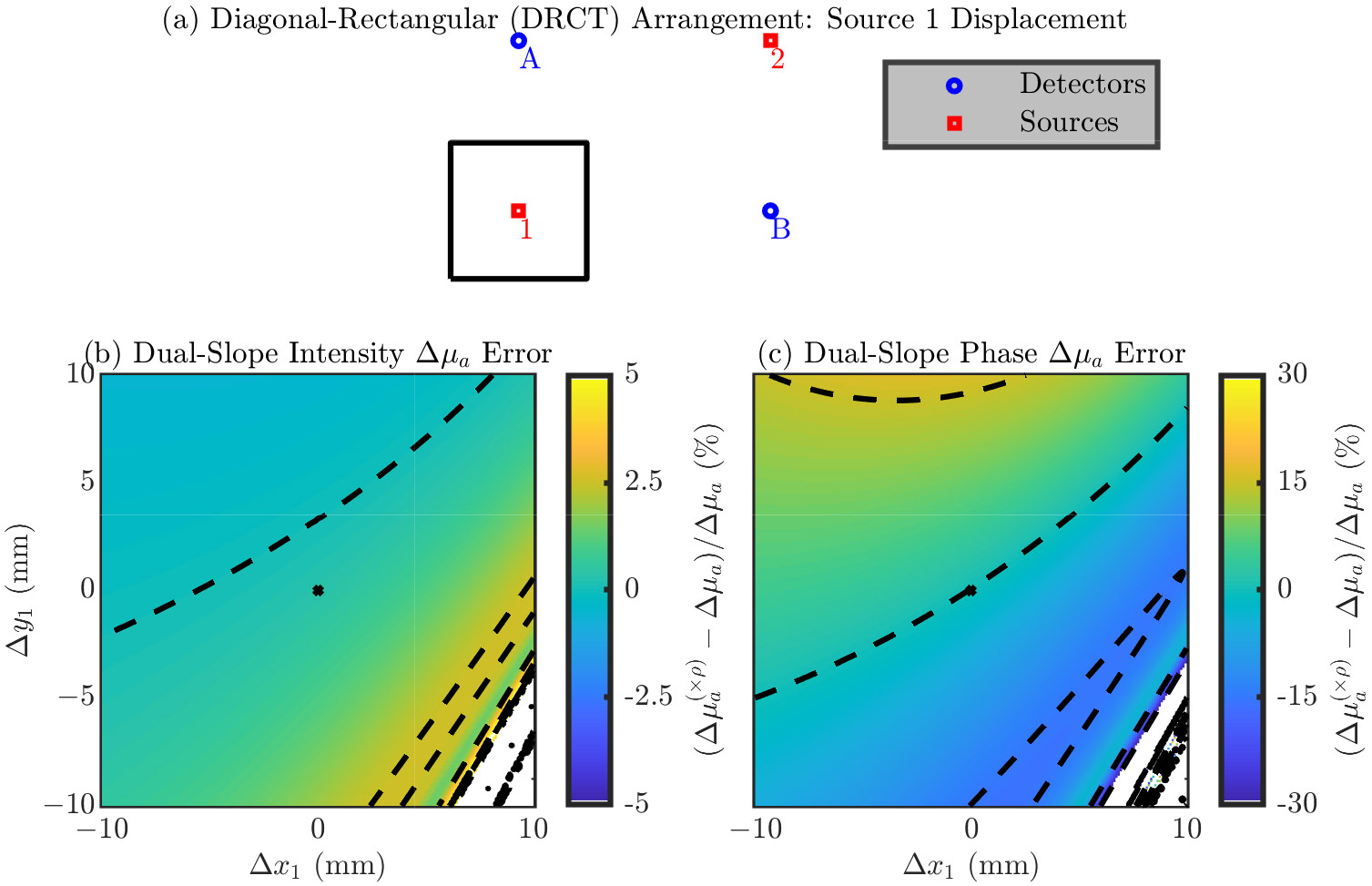
Error in the accuracy of recovering absorption coefficient change ($\Delta \mu_{a}$) for the Diagonal-ReCTangular (DRCT) arrangement. In the color maps the black dashed iso-lines represent the color bar tick-mark values and the nominal optode position is shown as a black asterisk. (**a**–**c**) Errors from displacement of source 1 (equivalent to source 2, detector A, and detector B). (**b**) Error in the $\Delta \mu_{a}$ recovered by Dual-Slope (DS) Intensity (I). (**c**) Error in the $\Delta \mu_{a}$ recovered by DS phase of photon density waves (ϕ). Symbols: Displacement of optode from the nominal position ([Δx, Δy]), optical property recovered with the optode displaced from the nominal position ((×ρ) in superscript).

In the following section the figures are in the same format as Section 3.1 but show results relevant to DS measurements of $\Delta \mu_{a}$ instead of SC measurements of the absolute $\mu_{a}$ and $\mu_{s}^{\prime }$. These results show errors in the recovered $\Delta \mu_{a}$ (shown with a (×ρ) in superscript to indicate that the nominal position is always assumed) relative to the true $\Delta \mu_{a}$ simulated in the medium. Using DS, $\Delta \mu_{a}$ can be recovered with either only I data or only ϕ data; therefore, results are shown for both cases. Figures 6-9 show these results for the LINR, the ALIN, the TRAP, and the DRCT arrangements, respectively. As before, we suggest that one focus on the direction of the change in the error and the magnitude of the range in the error from displacing an optode in a 20 mm × 20 mm square around its nominal position.

Summarizing these results, we note that, overall, the relative errors in $\Delta \mu_{a}$ due to optode position errors are less then those for absolute optical properties. For DS I the error is often on the order of 1% from moving the optode in the 20 mm × 20 mm square. However, for DS ϕ the order is 10%. Therefore, the ability for DS ϕ to accurately recover values of $\Delta \mu_{a}$ depends much more on the optode position than DS I by an order of magnitude.

## 4. Discussion

Based on the results in this work, we can provide guidance on what type of SC/DS arrangement is more robust against optode position errors. Additionally, we can provide guidance on what absolute optical property recovery method to use. Regarding the latter, we advocate for the iterative absolute optical property recovery method [16] on FD SC data. We justify this by comparing the accuracy errors of the iterative and slopes method in a case where the optode positions are assumed correctly (Table 1). Focusing on the error in the recovered $\mu_{a}$, the iterative method showed an average error of 6%, while the iterative method showed 0.01%, with a similar relationship for the error in $\mu_{s}^{\prime }$. Additionally, if we consider how robust these two methods are against optode position errors (Table 2), we see that the iterative method either performs the same as the slopes method or has a slight advantage. Therefore, we conclude that the iterative method is preferable over the slopes method.

For a comparison of the SC/DS arrangements, we can examine the results in both Sections 3.1 and 3.2. Considering all of these together, we conclude that arrangements typically have a critical optode type, and movement of the critical optode causes large errors in the recovered values. The critical optode type is characterized by the type of optodes placed near the arrangement’s line of symmetry or centroid (e.g., detectors A and B in LINR). This is because the position of an optode with this characteristic more heavily influences the difference between the short and long ρ’s in the SC/DS set, and this difference is known to be a critical parameter for these measurements [4]. The critical type may be either sources or detectors since sources and detectors may be interchanged (e.g., if sources and detectors are switched in LINR then sources 1 and 2 are critical). For LINR and ALIN the critical optodes are optodes A and B, while for TRAP the importance of optodes 1 and 2 and optodes A and B is more equally balanced but optodes A and B are still slightly more critical than optodes 1 and 2. The DRCT arrangement is not ideal since all the optodes’ positions are critical, since displacement of any optode is geometrically equivalent to the displacement of another. Additionally, the results in Table 6 further suggest that the TRAP arrangement may be more robust against multiple optode position errors than the other arrangements. A second conclusion is that the error in $\mu_{s}^{\prime }$ or $\Delta \mu_{a}$ recovered by DS ϕ is more dependent on the position of the critical optodes than $\mu_{a}$ or $\Delta \mu_{a}$ recovered by DS I, respectively.

Results in Section 3.1 unsurprisingly show that $\mu_{s}^{\prime }$ is more susceptible to errors, but from the recovery method itself and from optode position errors. This agrees with our experience working with experimental SC data [16]. Additionally, we have confirmed that the spatial dependence of the error on optode displacement behaves the same for experimental data as it does for both these simulations and the error ‘s order of magnitude agree. Specifically, if we consider an in vivo dataset [20] and assume incorrect optode displacements, we recover errors of the same order of magnitude and direction as the simulation results herein (results not included here for brevity). A more unexpected result is that from Section 3.2, which shows that $\Delta \mu_{a}$ recovered by DS ϕ is more susceptible to errors than DS I by an order of magnitude. This may have implications when comparing $\Delta \mu_{a}’s$ recovered by either DS I or DS ϕ since a small optode position error could propagate to almost no error in DS I’s results but a large error in DS ϕ’s results, making comparison of the data-types tenuous.

### Design of Optode Support Structure

**Figure 10.**
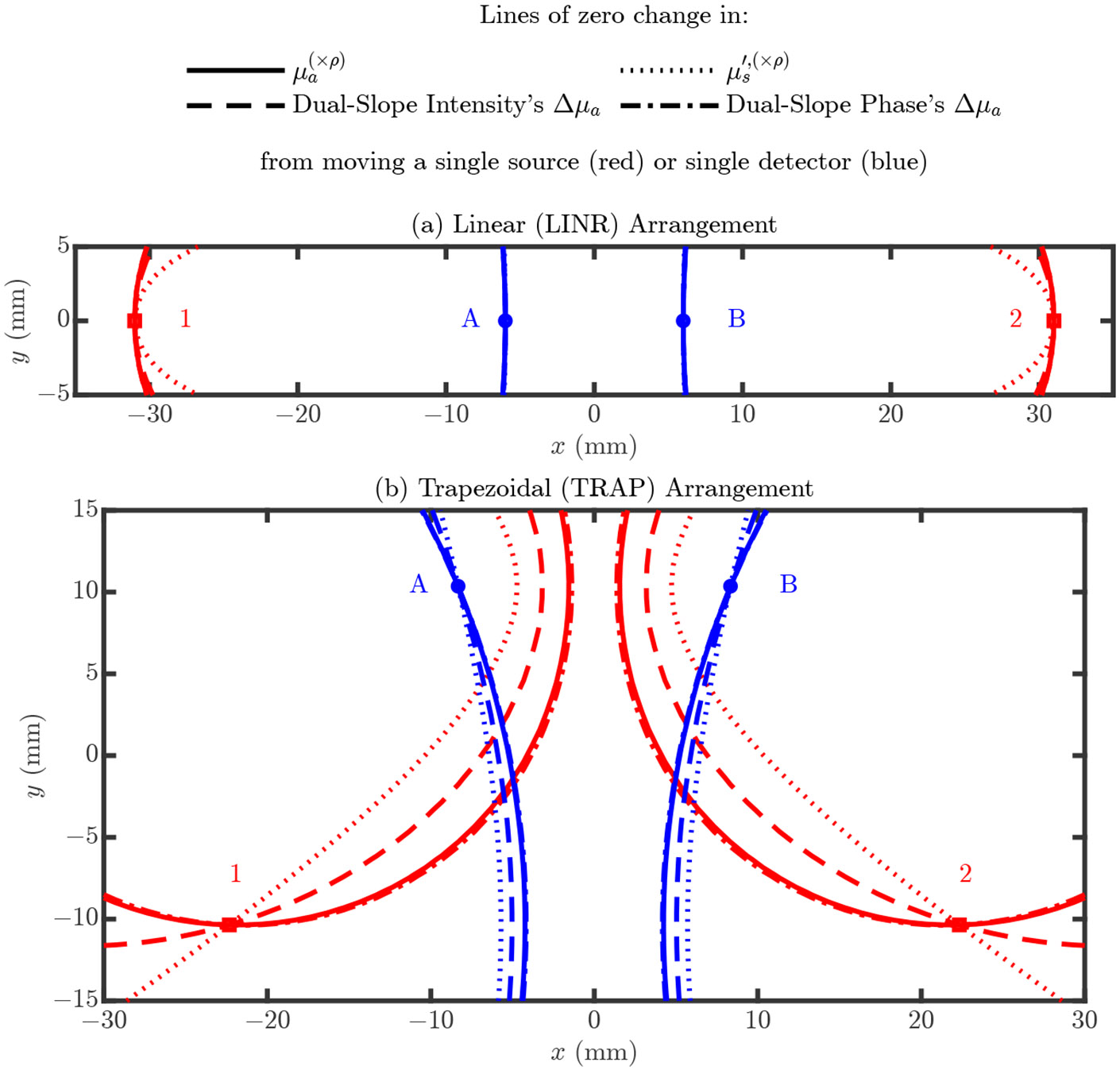
Lines for which, if the optode is moved along them, a recovered optical property will not change (i.e., lines of zero change), e.g., the solid red line which passes through source 1 shows positions where source 1 can be placed and the absorption coefficient ($\mu_{a}$) recovered by assuming the nominal ρ’s will be the same. (**a**) The LINeaR (LINR) arrangement. (**b**) The TRAPezoidal (TRAP) arrangement. Symbols: Reduced scattering coefficient ($\mu_{s}^{\prime }$), absorption coefficient change ($\Delta \mu_{a}$), optical property recovered with the optode displaced from the nominal position ((×ρ) in superscript).

The figures in Sections 3.1 and 3.2 reveal information about which optode displacement direction causes the largest error and which direction causes no change in the error. If an optode moves along its iso-line (i.e., line of constant error from the nominal optode position) in these figures, the recovered value will not change. Therefore, we wish to design a probe which prevents optode movement in directions which introduce errors but allows movement in directions that will not change the recovered value. Figure 10 shows these lines of zero change for the LINR and TRAP arrangements. These lines show positions where the optode can be placed that will not change the recovered value of a particular optical property. The lines for different optical properties intersect at the nominal optode position since this is the only position which will cause no error change for any of the optical properties. We can use Figure 10 to determine which directions it is allowable for optodes to move in (i.e., along the lines of zero change) in a real-life probe design.

**Figure 11.**
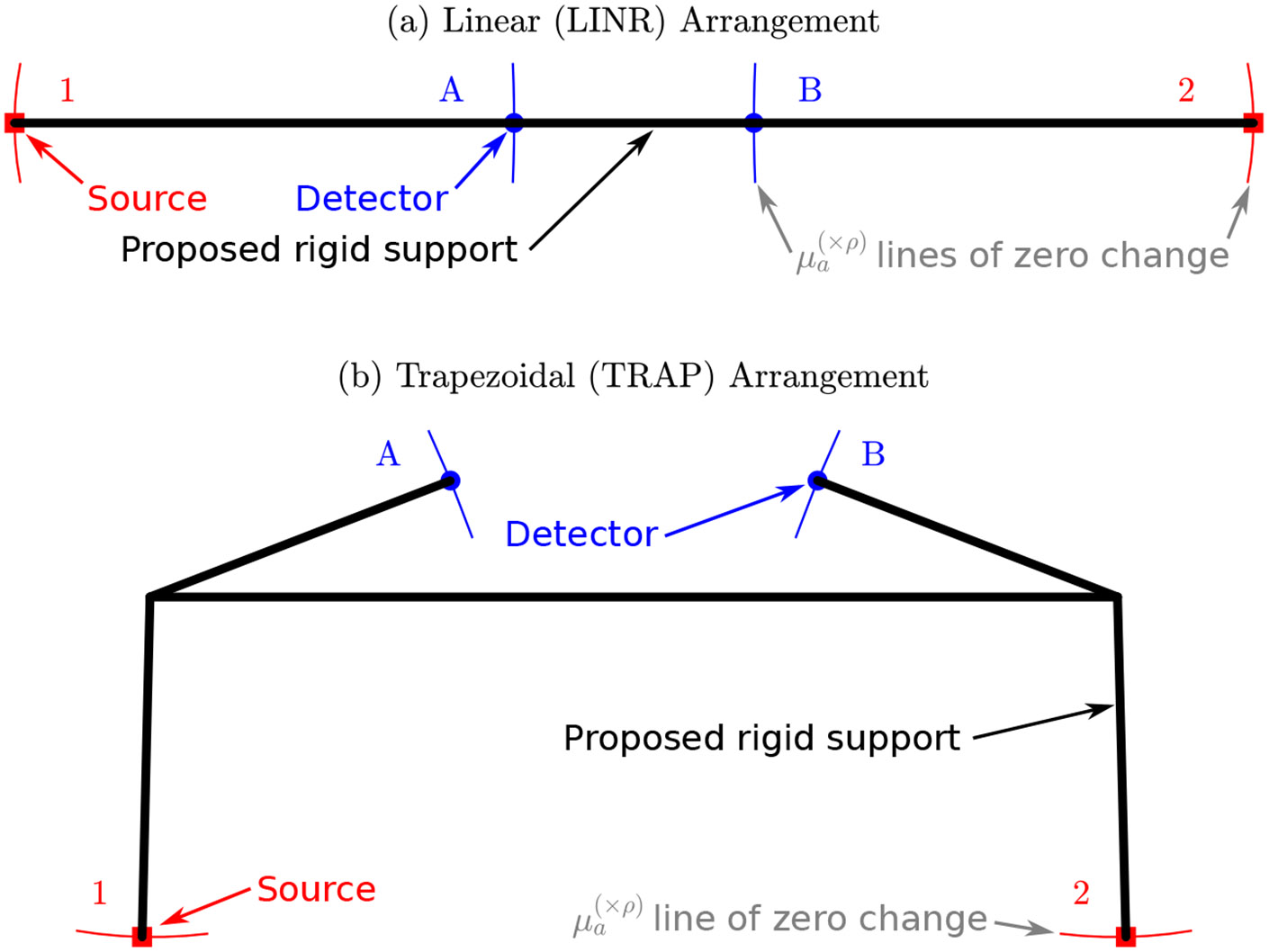
Proposed support structure, which was designed by allowing moving along the lines of zero change (Figure 10) but restricting movement perpendicular to the line. Lines in this figure considered zero change in absorption coefficient ($\mu_{a}$). (**a**) The LINeaR (LINR) arrangement. (**b**) The TRAPezoidal (TRAP) arrangement. Symbols: Optical property recovered with the optode displaced from the nominal position ((×ρ) in superscript).

Figure 11 shows how these lines of zero change can be used to design a DS/SC probe. Since movement along the line of zero change is allowable but movement perpendicular to that causes an error, one can design rigid supports which are perpendicular to the tangent of the line of zero change. We then extend these supports until they intersect to show the proposed skeleton of rigid supports. This proposed support structure should reduce errors induced by incorrect assumptions of the optode position, since they do not allow the optodes to move in the direction of maximum error. For the LINR arrangement (Figure 11a) these supports are trivial since they run along the linear line of the probe. However, for the TRAP arrangement (Figure 11b), this method of designing supports results in a non-obvious structure. This approach to design SC/DS probes will result in measurements more robust against optode position errors and probe deformation.

## 5. Conclusions

Self-calibrating and dual-slope measurements present a number of significant practical advantages by not requiring calibration of source emission and detector sensitivity [4], and by being relatively insensitive to movement artifacts [15]. These measurements rely on the fulfillment of specific geometrical requirements for the location of optodes [4,13] and knowledge of the location of all optodes. This work investigated the impact of uncertainties in the location of the optodes, which is especially relevant in the case of in vivo applications to biological tissues where the optical probe is required to conform to the shape of the investigated tissue. The significance of this work is twofold. First, it allows one to estimate the impact of uncertainties in the optode positions on absolute or relative optical measurements from continuous-wave or frequency-domain data in dual-slope configurations. Second, it guides the design of optical probes to minimize probe deformation along directions that result in optode displacements with the greatest impact on the optical measurements. Future work may include the investigation of other less impactful geometric sources of error for these diffuse optical measurements such as sample curvature (for relatively broad curvatures such as those relevant to the adult human head) or finite optode size.

## Figures and Tables

**Table 1.** Comparison of the accuracy of the slopes [2] versus the iterative [16] recovery method.

			Recovery Method			
ρ’s * (mm)	$\mu_{a}$ (mm^−1^)	$\mu_{s}^{\prime }$ (mm^−1^)	Slopes		Iterative	
			$\frac{\mu_{a}^{\left(\surd \rho \right)}-\mu_{a}}{\mu_{a}}\;(\%)$	$\frac{\mu_{s^{\prime }}^{\prime (\surd \rho )}-\mu_{s}^{\prime }}{\mu_{s}^{\prime }}\;(\%)$	$\frac{\mu_{a}^{\left(\surd \rho \right)}-\mu_{a}}{\mu_{a}}\;(\%)$	$\frac{\mu_{s^{\prime }}^{\prime (\surd \rho )}-\mu_{s}^{\prime }}{\mu_{s}^{\prime }}\;(\%)$
[25, 25, 37, 37]	0.005	0.5	11	−12	0.024	−0.99
	0.005	1.0	9.3	−2.9	0.0058	−0.50
	0.005	1.5	7.0	−1.2	0.0013	−0.33
	0.010	0.5	5.0	−12	0.029	−2.0
	0.010	1.0	4.7	−3.5	0.0038	−0.99
	0.010	1.5	3.6	−1.7	0.0046	−0.67
	0.015	0.5	2.9	−13	0.037	−3.0
	0.015	1.0	3.1	−4.0	0.0040	−1.5
	0.015	1.5	2.4	−2.1	0.0025	−1.00
[20, 30, 32, 42]	0.005	0.5	11	−13	0.025	−1.00
	0.005	1.0	9.7	−3.2	0.0070	−0.50
	0.005	1.5	7.4	−1.3	0.0016	−0.33
	0.010	0.5	4.7	−13	0.032	−2.0
	0.010	1.0	4.9	−3.8	0.0046	−0.99
	0.010	1.5	3.8	−1.8	0.0011	−0.66
	0.015	0.5	2.6	−14	0.041	−3.0
	0.015	1.0	3.3	−4.3	0.0048	−1.5
	0.015	1.5	2.6	−2.2	0.0030	−1.00

Symbols: Source-detector distance (ρ), absorption coefficient ($\mu_{a}$), reduced scattering coefficient ($\mu_{s}^{\prime }$), true optical property of the medium (μ), optical property recovered with the optode in the nominal position ((√ρ) in superscript). * [25, 25, 37, 37] corresponds to the LINR, TRAP, and DRCT arrangements; [20, 30, 32, 42] corresponds to the ALIN arrangement.

**Table 2.** Errors in the recovered optical properties from a 1 mm displacement in any direction relative to the value recovered in the nominal position, for the LINeR (LINR) arrangement.

			Recovery Method			
$\mu_{a}$ (mm^−1^)	$\mu_{s}^{\prime }$ (mm^−1^)	Optode	Slopes		Iterative	
			$\frac{\sigma_{\mu_{a}}^{\emptyset 2\text{mm}}}{\mu_{a}^{\left(\surd \rho \right)}}\;(\%)$	$\frac{\sigma_{\mu_{s}^{\prime }}^{\emptyset 2\text{mm}}}{\mu_{s^{\prime }}^{\prime (\surd \rho )}}\;(\%)$	$\frac{\sigma_{\mu_{a}}^{\emptyset 2\text{mm}}}{\mu_{a}^{\left(\surd \rho \right)}}\;(\%)$	$\frac{\sigma_{\mu_{s}^{\prime }}^{\emptyset 2\text{mm}}}{\mu_{s^{\prime }}^{\prime (\surd \rho )}}\;(\%)$
0.005	0.5	1 or 2	1.1	0.56	1.2	0.44
0.005	0.5	A or B	5.1	16	5.0	13
0.005	1.0	1 or 2	0.82	0.52	0.92	0.50
0.005	1.0	A or B	3.5	15	4.6	14
0.005	1.5	1 or 2	0.68	0.46	0.75	0.45
0.005	1.5	A or B	2.9	14	3.8	14
0.010	0.5	1 or 2	0.68	0.37	0.71	0.29
0.010	0.5	A or B	3.4	15	3.0	13
0.010	1.0	1 or 2	0.53	0.37	0.56	0.35
0.010	1.0	A or B	2.3	14	2.8	13
0.010	1.5	1 or 2	0.44	0.33	0.46	0.32
0.010	1.5	A or B	1.9	14	2.3	13
0.015	0.5	1 or 2	0.53	0.27	0.54	0.22
0.015	0.5	A or B	2.8	15	2.3	12
0.015	1.0	1 or 2	0.42	0.29	0.44	0.28
0.015	1.0	A or B	1.9	14	2.2	13
0.015	1.5	1 or 2	0.34	0.27	0.36	0.26
0.015	1.5	A or B	1.5	13	1.8	13

Symbols: Absorption coefficient ($\mu_{a}$), reduced scattering coefficient ($\mu_{s}^{\prime }$), root-mean-squared-error from orbiting the optode position around the nominal optode position in a 2 mm diameter circle ($\sigma^{\emptyset 2\;\text{mm}}$), optical property recovered with the optode in the nominal position ((√ρ) in superscript).

**Table 3.** Errors in the recovered optical properties from a 1 mm displacement in any direction relative to the value recovered in the nominal position, for the Asymmetric-LINear (ALIN) arrangement.

$\mu_{a}$ (mm^−1^)	$\mu_{s}^{\prime }$ (mm^−1^)	Optode	$\frac{\sigma_{\mu_{a}}^{\emptyset 2\;\text{mm}}}{\mu_{a}^{\left(\surd \rho \right)}}\;(\%)$	$\frac{\sigma_{\mu_{s}^{\prime }}^{\emptyset 2\;\text{mm}}}{\mu_{s^{\prime }}^{\prime (\surd \rho )}}\;(\%)$
0.005	0.5	1	1.5	0.56
0.005	0.5	2	0.88	0.34
0.005	0.5	A	5.3	13
0.005	0.5	B	4.6	13
0.005	1.0	1	1.3	0.69
0.005	1.0	2	0.67	0.37
0.005	1.0	A	5.0	14
0.005	1.0	B	4.3	14
0.005	1.5	1	1.1	0.64
0.005	1.5	2	0.54	0.33
0.005	1.5	A	4.2	14
0.005	1.5	B	3.7	14
0.010	0.5	1	0.95	0.37
0.010	0.5	2	0.54	0.23
0.010	0.5	A	3.2	13
0.010	0.5	B	2.8	12
0.010	1.0	1	0.81	0.48
0.010	1.0	2	0.41	0.26
0.010	1.0	A	3.1	13
0.010	1.0	B	2.7	13
0.010	1.5	1	0.68	0.45
0.010	1.5	2	0.34	0.24
0.010	1.5	A	2.6	14
0.010	1.5	B	2.3	13
0.015	0.5	1	0.73	0.28
0.015	0.5	2	0.42	0.18
0.015	0.5	A	2.4	12
0.015	0.5	B	2.1	12
0.015	1.0	1	0.63	0.38
0.015	1.0	2	0.32	0.21
0.015	1.0	A	2.4	13
0.015	1.0	B	2.1	13
0.015	1.5	1	0.52	0.37
0.015	1.5	2	0.26	0.19
0.015	1.5	A	2.0	13
0.015	1.5	B	1.7	13

Symbols: Absorption coefficient ($\mu_{a}$), reduced scattering coefficient ($\mu_{s}^{\prime }$), root-mean-squared-error from orbiting the optode position around the nominal optode position in a 2 mm diameter circle ($\sigma^{\emptyset 2\;\text{mm}}$), optical property recovered with the optode in the nominal position ((√ρ) in superscript).

**Table 4.** Errors in the recovered optical properties from a 1 mm displacement in any direction relative to the value recovered in the nominal position, for the TRAPezoidal (TRAP) arrangement.

$\mu_{a}$ (mm^−1^)	$\mu_{s}^{\prime }$ (mm^−1^)	Optode	$\frac{\sigma_{\mu_{a}}^{\emptyset 2\text{mm}}}{\mu_{a}^{\left(\surd \rho \right)}}\;(\%)$	$\frac{\sigma_{\mu_{s}^{\prime }}^{\emptyset 2\text{mm}}}{\mu_{s^{\prime }}^{\prime (\surd \rho )}}\;(\%)$
0.005	0.5	1 or 2	1.5	2.5
0.005	0.5	A or B	3.7	9.3
0.005	1.0	1 or 2	1.2	2.7
0.005	1.0	A or B	3.3	9.9
0.005	1.5	1 or 2	1.0	2.7
0.005	1.5	A or B	2.7	9.9
0.010	0.5	1 or 2	0.91	2.4
0.010	0.5	A or B	2.2	8.9
0.010	1.0	1 or 2	0.77	2.6
0.010	1.0	A or B	2.0	9.5
0.010	1.5	1 or 2	0.63	2.6
0.010	1.5	A or B	1.7	9.5
0.015	0.5	1 or 2	0.69	2.4
0.015	0.5	A or B	1.7	8.8
0.015	1.0	1 or 2	0.59	2.5
0.015	1.0	A or B	1.6	9.3
0.015	1.5	1 or 2	0.49	2.5
0.015	1.5	A or B	1.3	9.3

Symbols: Absorption coefficient ($\mu_{a}$), reduced scattering coefficient ($\mu_{s}^{\prime }$), root-mean-squared-error from orbiting the optode position around the nominal optode position in a 2 mm diameter circle ($\sigma^{\emptyset 2\;\text{mm}}$), optical property recovered with the optode in the nominal position ((√ρ) in superscript).

**Table 5.** Errors in the recovered optical properties from a 1 mm displacement in any direction relative to the value recovered in the nominal position, for the Diagonal-ReCTangular (DRCT) arrangement.

$\mu_{a}$ (mm^−1^)	$\mu_{s}^{\prime }$ (mm^−1^)	Optode	$\frac{\sigma_{\mu_{a}}^{\emptyset 2\;\text{mm}}}{\mu_{a}^{\left(\surd \rho \right)}}\;(\%)$	$\frac{\sigma_{\mu_{s}^{\prime }}^{\emptyset 2\;\text{mm}}}{\mu_{s^{\prime }}^{\prime (\surd \rho )}}\;(\%)$
0.005	0.5	1, 2, A, or B	3.7	9.3
0.005	1.0	1, 2, A, or B	3.3	9.9
0.005	1.5	1, 2, A, or B	2.7	9.9
0.010	0.5	1, 2, A, or B	2.2	8.9
0.010	1.0	1, 2, A, or B	2.0	9.5
0.010	1.5	1, 2, A, or B	1.7	9.5
0.015	0.5	1, 2, A, or B	1.7	8.8
0.015	1.0	1, 2, A, or B	1.6	9.3
0.015	1.5	1, 2, A, or B	1.3	9.3

Symbols: Absorption coefficient ($\mu_{a}$), reduced scattering coefficient ($\mu_{s}^{\prime }$), root-mean-squared-error from orbiting the optode position around the nominal optode position in a 2 mm diameter circle ($\sigma^{\emptyset 2\;\text{mm}}$), optical property recovered with the optode in the nominal position ((√ρ) in superscript).

**Table 6.** Errors in the recovered optical properties from a simultaneous 1 mm displacement by multiple optodes in any direction relative to the value recovered in the nominal positions.

Arrangement	Optodes	$\frac{\sigma_{\mu_{a}}^{\emptyset 2\;\text{mm}}}{\mu_{a}^{\left(\surd \rho \right)}}\;(\%)$	$\frac{\sigma_{\mu_{s}^{\prime }}^{\emptyset 2\;\text{mm}}}{\mu_{s^{\prime }}^{\prime (\surd \rho )}}\;(\%)$
LINR	1 and A	2.9	13
	1 and B	2.9	13
	1 and 2	0.80	0.49
	A and B	4.0	19
	1, A, and B	4.0	19
	1, 2, and A	2.9	13
	1, 2, A, and B	4.1	19
ALIN	1 and A	3.2	13
	1 and B	2.8	13
	2 and A	3.1	13
	2 and B	2.7	13
	1 and 2	0.91	0.55
	A and B	4.1	19
	1, A, and B	4.2	19
	2, A, and B	4.1	19
	1, 2, and A	3.2	13
	1, 2, and B	2.8	13
	1, 2, A, and B	4.2	19
TRAP	1 and A	2.2	9.8
	1 and B	2.2	9.8
	1 and 2	1.1	3.6
	A and B	2.9	13
	1, A, and B	3.0	14
	1, 2, and A	2.3	10
	1, 2, A, and B	3.1	14
DRCT	1 and A	2.9	13
	1 and B	2.9	13
	1 and 2	2.9	13
	1, A, and B	3.5	16
	1, 2, A, and B	4.1	19

Symbols: Absorption coefficient ($\mu_{a}$), reduced scattering coefficient ($\mu_{s}^{\prime }$), root-mean-squared-error from orbiting the optode position around the nominal optode position in a 2 mm diameter circle ($\sigma^{\emptyset 2\;\text{mm}}$), optical property recovered with the optode in the nominal position ((√ρ) in superscript).

## Data Availability

Supporting code for this manuscript can be found at the following link: https://github.com/DOIT-Lab/DOIT-Public/tree/master/SelfCalibratingPositionErrors accessed on 9 July 2025.
